# Dynamic Adsorption of Mn^2+^ from Acid Mine Drainage by Highly Active Immobilized Particles with Fe^0^/Fe^2+^ Enhanced SRB

**DOI:** 10.3390/molecules29184497

**Published:** 2024-09-22

**Authors:** He Chen, Laigui Wang, Wenbo An, Qiqi Wang

**Affiliations:** 1 School of Mechanics and Engineering, Liaoning Technical University, Fuxin 123000, China; wanglaigui123@163.com; 2 School of Civil Engineering, Liaoning Technical University, Fuxin 123000, China; wb_an1992@163.com (W.A.); wangqiqi99825@163.com (Q.W.)

**Keywords:** acid mine drainage, Fe^0/2+^-SRB immobilized particles, dynamic adsorption, sulfate-reducing bacteria, heavy metals

## Abstract

Bioremediation of acid mine drainage (AMD) was often challenged by poor tolerance of sulfate-reducing bacteria (SRB) to heavy metals and low bioactivity. The highly active immobilized particles with Fe^0^/Fe^2+^ enhanced SRB (Fe^0/2+^-SRB) were prepared by the microorganism immobilization technique. Three dynamic columns were constructed to investigate the adsorption capacity of Fe^0/2+^-SRB for Mn^2+^ under varying adsorption layer heights, inflow velocity, and initial Mn^2+^ concentrations. The role of each matrix material in the immobilized particles was explored, the mechanism of AMD remediation by Fe^0/2+^-SRB was revealed, and the adaptability of Fe^0/2+^-SRB to AMD under various initial conditions was investigated. The results showed that the prepared Fe^0/2+^-SRB exhibited a well-developed surface pore structure. When the adsorption layer height was 200 mm, the influent flow rate was 5 × 10^−5^ m^3^/s, and the initial manganese ion concentration was 10 mg/L, the maximum dynamic adsorption capacities (*q*_e_) of Mn^2+^ for each dynamic column were 7.8430, 4.7627, and 8.7677 mg/g, respectively. Compared to dynamic columns 1# and 2#, dynamic column 3# showed the best performance in treating AMD, and the Thomas model effectively described the adsorption kinetics of Mn^2+^ by Fe^0/2+^-SRB(3#). Microstructural analysis indicated that chemical adsorption, ion exchange, dissimilation–reduction reaction, and surface complexation occurred between the various matrix materials in Fe^0/2+^-SRB(3#). Mn^2+^ was primarily removed in the form of metal sulfide (MnS), and Fe^0^/Fe^2+^ could promote the dissimilatory reduction of SO_4_^2−^ by SRB to form S^2−^. Fe^0/2+^-SRB(3#) was able to adapt to AMD with initial conditions of pH was 2~4, SO_4_^2−^ < 2500 mg/L, and Mn^2+^ < 20 mg/L. The research results provide new insights into the remediation of AMD, using a combined microbial-adsorption technology.

## 1. Introduction

Acid mine drainage (AMD) is the primary environmental pollution problem faced by the global mining industry [1]. It is a type of wastewater characterized by high acidity, high concentrations of heavy metals, and high concentrations of sulfates (collectively referred to as “three highs” wastewater), generated when sulfide minerals in coal seams and surrounding rocks come into contact with oxygen and water during coal resource extraction and utilization [2,3,4]. Once AMD is generated, it contaminates surrounding water and soil, rendering them toxic and passing this toxicity to the flora and fauna that inhabit these environments. Ultimately, human health is significantly threatened by the biomagnification effect in the food chain [5]. In recent years, AMD has had relatively negative impacts on many countries around the world, including China, the United States, Canada, Australia, and South Africa [6]. Globally, there are between 20,000 and 50,000 mines generating AMD, contaminating 19,300 square kilometers of freshwater and 7.2 million square kilometers of lakes and reservoirs [7]. Therefore, it is especially important to seek a socially, economically, and environmentally sustainable method for AMD remediation.

The microbial method has gained widespread attention from scholars both domestically and internationally due to its low cost, strong adaptability, absence of secondary pollution, and the ability to recover heavy metals in the form of sulfide precipitates [8,9,10]. This method primarily utilizes sulfate-reducing bacteria (SRB) in anaerobic environments, where they release electrons through a series of biological oxidation processes to reduce SO_4_^2−^ to S^2−^. The S^2−^ can bind with heavy metal ions to form insoluble sulfides, effectively removing the heavy metals, while also combining with the abundant H^+^ in the water to form H_2_S gas, which exits the system, thereby consuming H^+^ and increasing the pH [11,12,13]. The key to the successful remediation of AMD using the microbial method lies in providing sufficient carbon sources and a suitable growth environment to maintain SRB activity [14]. However, most AMD contains very limited carbon sources (<3–4 mg/L) [15], which are insufficient to support the active growth and metabolic activity of SRB. Additionally, the high acidity of AMD can further increase the solubility of certain heavy metal ions, exacerbating the harsh “three highs” environment and inhibiting SRB activity [16]. Therefore, to ensure the biological activity of SRB and to protect them from the toxic effects of AMD, microbial immobilization technology has emerged as a method for AMD remediation [17]. Zhang et al. [18,19] prepared immobilized SRB microbeads that exhibited tolerance to AMD with high concentrations of heavy metals. The maximum sulfate removal rate reached 2.67 g/(L d), and the removal rates of heavy metals in AMD under acidic conditions (pH 2.8) were above 99.9% (except for manganese, which ranged from 42.1% to 99.3%). Therefore, embedding SRB and inexpensive carbon sources into the same immobilized particles is feasible, but ensuring the longevity and efficiency of SRB activity remains crucial. An et al. [20] found that Fe^0^ undergoes corrosion in AMD, converting H^+^ into H_2_. The H_2_ produced serves as an electron donor for SRB, while the Fe^2+^ generated is an active component of various enzymes within SRB cells, acting as an activator for the enzyme that catalyzes the reduction of SO_4_^2−^. Additionally, maifan stone exhibits strong adsorption capabilities for heavy metals through ion exchange and surface complexation reactions. To date, most studies [14,21] have used static batch adsorption experiments, which are suitable only for treating small volumes of wastewater and do not fully represent real-world conditions, thus presenting certain limitations. In practical remediation processes, large volumes of AMD wastewater often flow dynamically under continuous conditions, making the study of dynamic adsorption characteristics highly valuable and significant for real-world engineering applications.

In this study, microbial immobilization technology was adopted. The iron powders, corn cobs, and maifan stone serve as the primary matrix materials, which are organically combined with SRB. The materials were embedded to prepare highly active immobilized particles with Fe^0^/Fe^2+^ enhanced SRB. The adaptability of these SRB immobilized particles to AMD under various initial conditions was analyzed. Three dynamic columns were constructed: 1# (filled with immobilized particles without iron powder), 2# (filled with immobilized particles containing only iron powder), and 3# (filled with immobilized particles containing all materials) were constructed. The roles of various matrix materials within the immobilized particles were explored. The mechanisms by which the Fe^0^/Fe^2+^ composite system enhances SRB remediation of acid mine drainage were also elucidated. The aim is to provide a novel adsorbent for the microbial treatment of AMD in mining areas, thereby addressing the gaps in understanding the patterns and mechanisms of dynamic adsorption.

## 2. Results and Discussion

### 2.1. Dynamic Adsorption Performance of Fe^0/2+^-SRB for Mn^2+^

#### 2.1.1. Breakthrough Curves of Fe^0/2+^-SRB for Mn^2+^ at Different Adsorption Layer Heights

**Figure 1:**
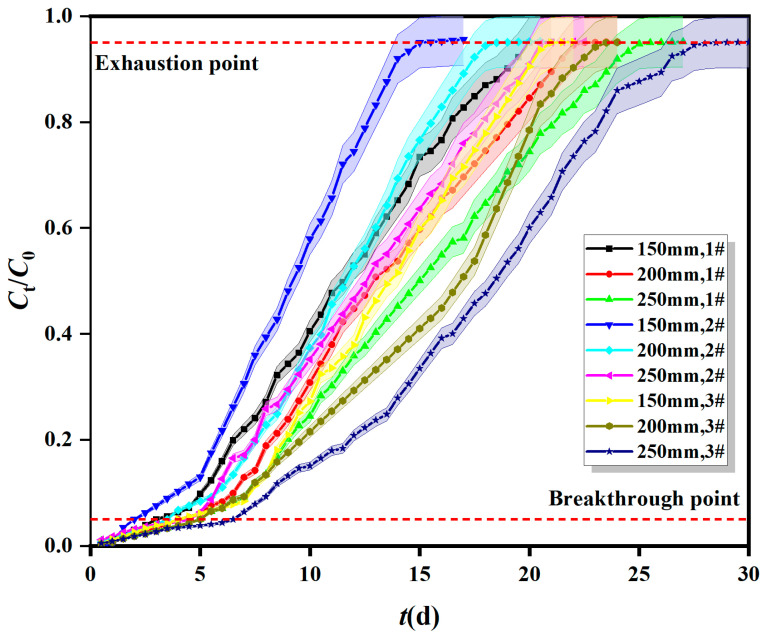
Breakthrough curves of Fe^0/2+^-SRB for Mn^2+^ at different adsorption layer heights in dynamic columns.

The breakthrough curves of Fe^0/2+^-SRB for Mn^2+^ at different adsorption layer heights in dynamic columns (1#, 2#, and 3#) are shown in Figure 1. The initial influent flow rate was 5 × 10^−5^ m^3^/s and the initial Mn^2+^ concentration was 10 mg/L. The heights of the adsorption layers (Fe^0/2+^-SRB mass) were 150 mm (300 g), 200 mm (470 g), and 250 mm (610 g). The adsorption mass transfer parameters of Fe^0/2+^-SRB for Mn^2+^ at different adsorption layer heights in dynamic columns are shown in Table 1.

As shown in Figure 1, the Mn^2+^ breakthrough curves for dynamic columns 1#, 2#, and 3# shift from left to right with increasing adsorption layer height (Fe^0/2+^-SRB mass). The slope gradually increases, and both the breakthrough and exhaustion points are delayed sequentially, extending the time to reach dynamic adsorption equilibrium. The breakthrough curves follow the ideal “S” type adsorption characteristic. Specifically, as the adsorption layer height increases, the surface area of Fe^0/2+^-SRB increases, the number of active sites on the particle surface increases, and the mass transfer zone length increases, thus prolonging the adsorption time [22]. Table 1 shows that the total adsorption amount (*q*_total_) is positively correlated with the adsorption layer height. When the adsorption layer height decreases, the spatial resistance of AMD flowing through the dynamic column reduces, resulting in a shorter contact time between Mn^2+^ and Fe^0/2+^-SRB, so Mn^2+^ cannot be completely adsorbed by Fe^0/2+^-SRB. Conversely, when the adsorption layer height increases, the contact time between Mn^2+^ and Fe^0/2+^-SRB extends, allowing Mn^2+^ to be better adsorbed by Fe^0/2+^-SRB, which increases the adsorption amount [23]. However, the adsorption capacity (*q*_e_) is negatively correlated with the adsorption layer height. When the Fe^0/2+^-SRB mass is too high, excessive crowding between particles occurs, leading to overlapping and coverage of active sites on the Fe^0/2+^-SRB particles. As AMD flows through the dynamic column, it often exits before reaching the maximum saturation capacity. The higher the adsorption layer height, the larger the unsaturated portion, resulting in a decrease in adsorption capacity (*q*_e_) with increasing adsorption layer height.

#### 2.1.2. Breakthrough Curves of Fe^0/2+^-SRB for Mn^2+^ at Different Inflow Velocity

**Figure 2:**
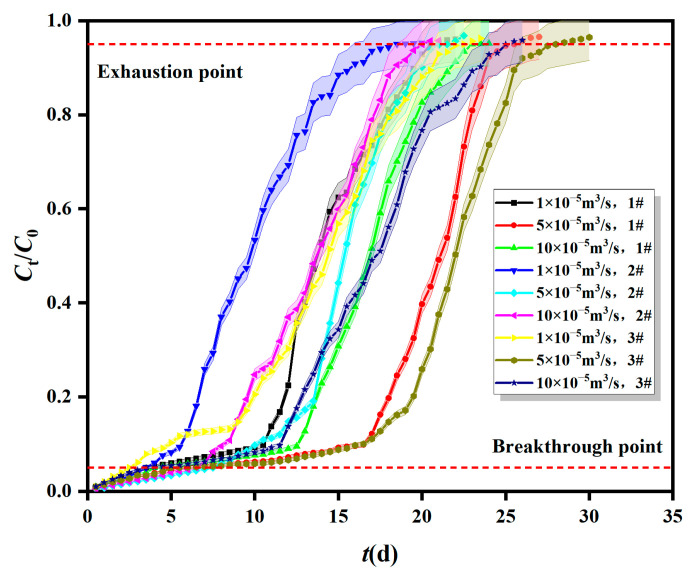
Breakthrough curves of Fe^0/2+^-SRB for Mn^2+^ at different inflow velocities.

The breakthrough curves of Fe^0/2+^-SRB for Mn^2+^ at different inflow velocities are shown in Figure 2. The initial Mn^2+^ concentration was 10 mg/L, and the adsorption layer height (Fe^0/2+^-SRB mass) was 200 mm (470 g). The initial velocities were 1 × 10^−5^ m^3^/s, 5 × 10^−5^ m^3^/s, and 10 × 10^−5^ m^3^/s. The adsorption mass transfer parameters for Fe^0/2+^-SRB for Mn^2+^ at different inflow velocities are shown in Table 1.

As shown in Figure 2, the membrane mass transfer resistance of Mn^2+^ through Fe^0/2+^-SRB particles decreases as the flow rate increases, reducing the residence time of AMD in the dynamic column, and thus, the adsorption performance of Fe^0/2+^-SRB for Mn^2+^ deteriorates [24]. From Table 1, it can be seen that the maximum values of total adsorption capacity (*q*_total_) and adsorption capacity (*q*_e_) of Fe^0/2+^-SRB for Mn^2+^ occur at an inlet flow rate of 5 × 10^−5^ m^3^/s, which is between 1 × 10^−5^ m^3^/s and 10 × 10^−5^ m^3^/s. This suggests that both excessively high and low velocities are detrimental to the dynamic adsorption of Mn^2+^ by Fe^0/2+^-SRB. If the inlet flow rate is too high, the residence time of AMD in the dynamic column is shorter, resulting in less contact time between AMD and Fe^0/2+^-SRB, which is unfavorable for the diffusion and adsorption of Mn^2+^, thus reducing the mass transfer efficiency of Fe^0/2+^-SRB. Conversely, if the inlet flow rate is too low, AMD remains in the dynamic column longer, allowing more time for Mn^2+^ to diffuse into the Fe^0/2+^-SRB particles. This improves the adsorption performance for Mn^2+^, but the volume of AMD treated per unit time decreases. Additionally, a lower flow rate may lead to vertical back-mixing of the liquid phase in the dynamic column, reducing the effective utilization of Fe^0/2+^-SRB.

#### 2.1.3. Breakthrough Curves of Fe^0/2+^-SRB for Mn^2+^ at Different Initial Concentrations

**Figure 3:**
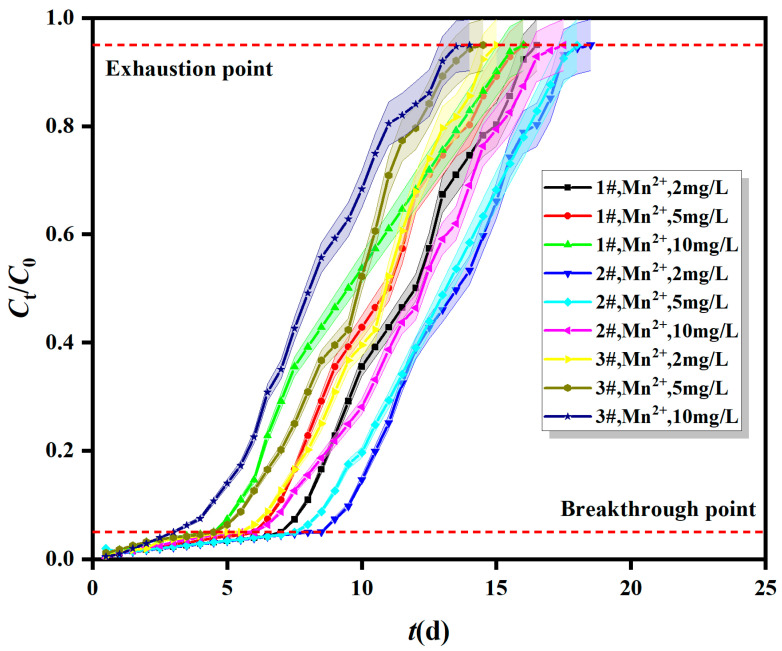
Breakthrough curves of Fe^0/2+^-SRB for Mn^2+^ at different initial concentrations.

The breakthrough curves of Fe^0/2+^-SRB for Mn^2+^ at different initial concentrations are shown in Figure 3. The dynamic column adsorption layer height (Fe^0/2+^-SRB mass) was 200 mm (470 g) and the inlet flow rate was 5 × 10^−5^ m^3^/s. The initial concentrations of Mn^2+^ were 2 mg/L, 5 mg/L, and 10 mg/L. The adsorption mass transfer parameters for Fe^0/2+^-SRB for Mn^2+^ at different initial concentrations are shown in Table 1.

Figure 3 and Table 1 show that the total adsorption quantity (*q*_total_) and the adsorption capacity (*q*_e_) of Fe^0/2+^-SRB for Mn^2+^ increase with higher initial concentrations. Higher initial concentrations of Mn^2+^ provide a greater driving force for the mass transfer of Mn^2+^ to Fe^0/2+^-SRB particles, enhancing the activation energy of the adsorption reaction [25]. This increases the transfer efficiency of Mn^2+^, allowing it to occupy the adsorption sites on Fe^0/2+^-SRB particles more quickly and accelerating the adsorption rate. At the same time, more Mn^2+^ comes into contact with Fe^0/2+^-SRB per unit time, making it easier to reach adsorption saturation. However, the total number of adsorption sites on Fe^0/2+^-SRB particles is limited, causing the breakthrough and exhaustion points to occur earlier and reducing the time required to achieve dynamic adsorption equilibrium, making breakthrough more likely [26]. However, lower initial Mn^2+^ concentrations result in decreased mass transfer efficiency, with lower diffusion and mass transfer coefficients. Therefore, higher initial concentrations are necessary to ensure the effective operation of the dynamic column [27].

Based on Table 1, under varying conditions of adsorption layer height, inflow velocity, and initial concentration, the total adsorption quantity (*q*_total_) and adsorption capacity (*q*_e_) of Fe^0/2+^-SRB for Mn^2+^ exhibit the following trend: 3# dynamic column > 1# dynamic column > 2# dynamic column. Fe^0/2+^-SRB(2#) particles contain only iron powder and perlite. Perlite has a loose, porous structure with a large surface area and strong chemical adsorption capabilities. In aqueous environments, perlite can release trace elements such as Al^3+^ and Fe^2+^, which can undergo ion exchange with Mn^2+^. Therefore, the adsorption of Mn^2+^ by Fe^0/2+^-SRB(2#) particles relies mainly on the ion exchange properties of perlite, while the influence of the Fe^0^/Fe^2+^ system formed by the iron powder on Mn^2+^ adsorption is minimal. Fe^0/2+^-SRB(1#) particles contain SRB, corn cobs, and perlite. SRB can reduce SO_4_^2−^ in AMD under anaerobic conditions, and the generated S^2−^ reacts with Mn^2+^ to form metal sulfide MnS through surface complexation. Corn cobs serve as a carbon source to support the dissimilatory reduction by SRB. Therefore, the adsorption of Mn^2+^ by Fe^0/2+^-SRB(1#) particles involves not only the ion exchange by perlite but also the dissimilatory reduction and surface complexation by SRB. Fe^0/2+^-SRB(3#) particles, which contain iron powder, SRB, corn cobs, and perlite, show significantly higher total adsorption quantity (*q*_total_) and adsorption capacity (*q*_e_) for Mn^2+^ compared to Fe^0/2+^-SRB(2#) and Fe^0/2+^-SRB(1#). This is likely due to the presence of an “active iron” system formed by Fe^0^/Fe^2+^ in the acidic, anaerobic environment. Whether this system can synergistically enhance SRB dissimilatory reduction requires further microscopic investigation.

### 2.2. Dynamic Adsorption Kinetics Models of Fe^0/2+^-SRB for Mn^2+^

Taking the 3# dynamic column as an example, the breakthrough curve experimental data for Fe^0/2+^-SRB adsorption of Mn^2+^ were fitted using the Bohart–Adams model and the Thomas model (Table 2). This analysis provides insights into the dynamic adsorption process of Fe^0/2+^-SRB for Mn^2+^ under varying conditions of adsorption layer height, inflow velocity, and initial concentration of Mn^2+^ from a kinetic perspective.

According to Table 2, for the Bohart–Adams model, as the adsorption layer height increases from 150 mm to 250 mm, the Bohart–Adams mass transfer rate constant *k*_AB_ decreases, and the saturation concentration of Mn^2+^ in the effluent *N*_0_ decreases as well [28]. When the inflow rate increases from 1 × 10^−5^ m^3^/s to 10 × 10^−5^ m^3^/s, both *k*_AB_ and *N*_0_ initially increase and then decrease, with a maximum value at a flow rate of 5 × 10^−5^ m^3^/s, where *N*_0_ is 8.259 mg/L. This indicates that the initial adsorption kinetics of Fe^0/2+^-SRB for Mn^2+^ is controlled by external mass transfer [29]. When the initial concentration of Mn^2+^ increases from 2 mg/L to 10 mg/L, *k*_AB_ decreases, while *N*_0_ increases. The fitting parameters of the Bohart–Adams model are consistent with the trends observed in the breakthrough curves from Figure 1, Figure 2 and Figure 3. However, the correlation coefficient *R*^2^_AB_ ranges from 0.825 to 0.955, indicating that the Bohart–Adams model is not well-suited for describing the dynamic adsorption process of Fe^0/2+^-SRB for Mn^2+^. This model is typically used for describing the initial stage of breakthrough curves, assumes instantaneous adsorption equilibrium, and neglects internal mass transfer and membrane diffusion resistance.

**Figure 4:**
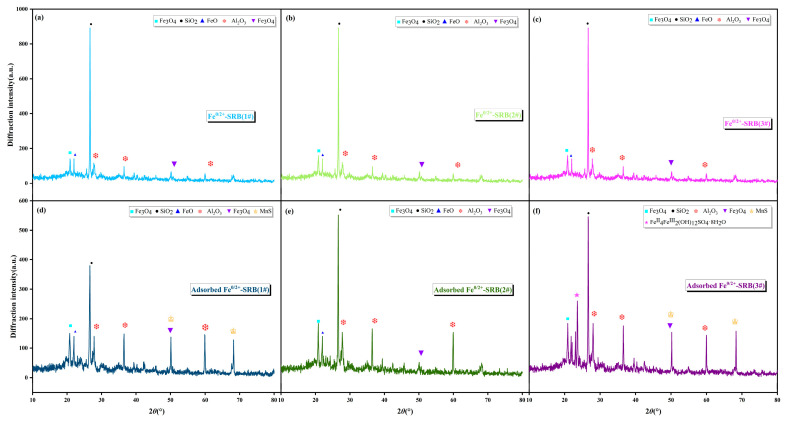
The results of X-ray diffraction (**a**) Fe^0/2+^-SRB(1#), (**b**) Fe^0/2+^-SRB(2#), (**c**) Fe^0/2+^-SRB(3#), (**d**) Adsorbed Fe^0/2+^-SRB(1#), (**e**) Adsorbed Fe^0/2+^-SRB(2#), (**f**) Adsorbed Fe^0/2+^-SRB(3#).

**Figure 5:**
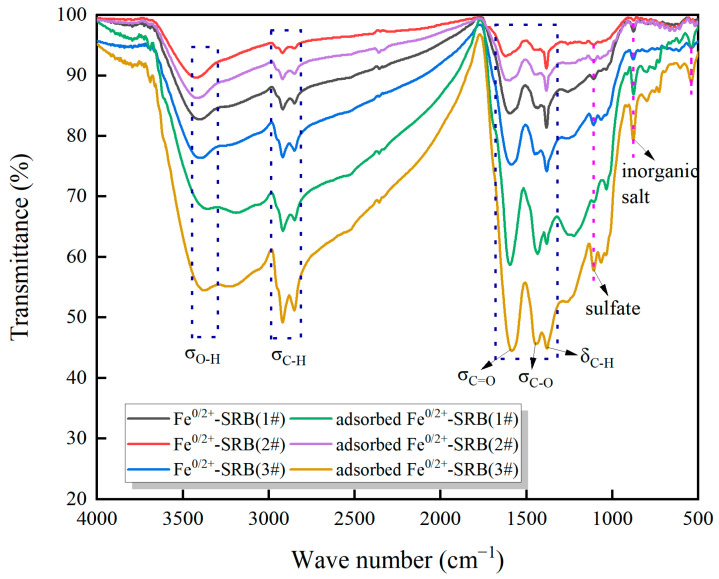
The results of Fourier transform infrared spectroscopy.

**Figure 6:**
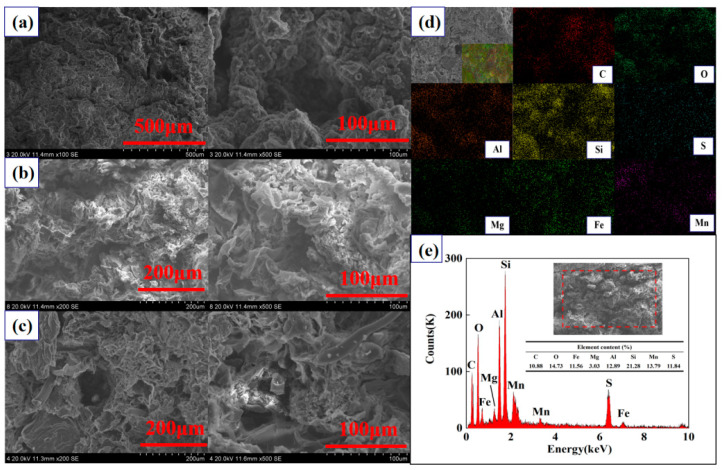
(**a**) SEM image of Fe^0/2+^-SRB(3#). (**b**,**c**) SEM images of adsorbed Fe^0/2+^-SRB(3#). (**d**) Selected area of adsorbed Fe^0/2+^-SRB(3#) for EDS analysis. (**e**) Elemental content of the selected area in the EDS analysis.

For the Thomas model, as the adsorption layer height increases from 150 mm to 250 mm, the Thomas mass transfer rate constant *k*_Th_ and the maximum adsorption capacity *q*_0_ of Fe^0/2+^-SRB both decrease. This indicates that increasing the adsorption layer height can increase the number of adsorption sites on Fe^0/2+^-SRB particles and prolong the overall operation time of the dynamic column. When the inflow rate increases from 1 × 10^−5^ m^3^/s to 10 × 10^−5^ m^3^/s, both *k*_Th_ and *q*_0_ first increase and then decrease, with *q*_0_ reaching a maximum value of 9.054 mg/g at an inflow rate of 5 × 10^−5^ m^3^/s. This suggests that an excessive flow rate can shorten the contact time between Mn^2+^ and Fe^0/2+^-SRB, reducing the utilization of active sites on Fe^0/2+^-SRB particles. As the initial concentration of Mn^2+^ increases from 2 mg/L to 10 mg/L, *k*_Th_ decreases while *q*_0_ increases. This indicates that an increased concentration gradient between Mn^2+^ and Fe^0/2+^-SRB enhances the driving force for mass transfer, making Mn^2+^ more readily adsorbed [30]. Similarly, the fitting parameters of the Thomas model are consistent with the trends observed in the breakthrough curves from Figure 4, Figure 5 and Figure 6. However, the correlation coefficient *R*^2^_Th_ for the Thomas model ranges from 0.978 to 0.995, indicating that the Thomas model provides a better description of the dynamic adsorption process of Fe^0/2+^-SRB for Mn^2+^ compared to the Bohart–Adams model. The dynamic adsorption process of Mn^2+^ using activated carbon particles prepared with mango dry as the matrix component can also be described using the Thomas model, as reported by Chowdhury [31]. Similarly, Naghmeh Fallah [32] used molybdenum (VI) as the template ion, isonicotinic acid (IN) as the functional monomer, and silica as the carrier to remove molybdenum ions. The surface imprinting method was employed to synthesize molybdenum (VI) ion-imprinted polymers (Mo(VI)-IIP) for removing molybdenum (VI) from aqueous solutions, resulting in similar findings.

### 2.3. Microscopic Characterization of Fe^0/2+^-SRB

#### 2.3.1. X-ray Diffraction Analysis

X-ray diffraction was used to analyze the changes in the composition of immobilized particles in dynamic columns 1# through 3# before and after adsorbing AMD under specific operating conditions, as shown in Figure 4. The specific operating conditions were as follows: dynamic column adsorbent layer height was 200 mm, inflow velocity was 5 × 10^−5^ m^3^/s, and initial concentration of Mn^2+^ was 10 mg/L. Figure 4 showed that before treating AMD, Fe^0/2+^-SRB(1#), Fe^0/2+^-SRB(2#), and Fe^0/2+^-SRB(3#) primarily contain four characteristic peaks corresponding to SiO_2_, FeO, Fe_3_O_4_, and Al_2_O_3_. These components mainly originate from the internal matrix materials of the immobilized particles (e.g., Maifan stone, iron powder) [33,34,35]. There are two peaks near 2*θ* = 50°, one is the characteristic peak of Fe_2_O_3_ at 49.49° (PDF#00-001-1053), and the other is the characteristic peak of MnS at 49.79° (PDF# 00-003-1062). After treating AMD, adsorbed Fe^0/2+^-SRB(1#) shows the presence of characteristic peaks for MnS, indicating that S^2−^ generated from the dissimilatory reduction of SO_4_^2−^ by SRB reacts with Mn^2+^ to form the metal sulfide MnS. The adsorbed Fe^0/2+^-SRB(2#) did not show the characteristic peak of MnS, indicating that the iron powder in the particles only promoted the dissimilar reduction of SRB, but did not play the role of adsorption and ion exchange. In contrast, adsorbed Fe^0/2+^-SRB(3#) also shows the characteristic peak for Fe^II^_4_Fe^III^_2_(OH)_12_SO_4_·8H_2_O. This is because Fe^0^, when present in an anaerobic environment, reacts with H^+^ in AMD to release Fe^2+^, increasing the relative concentration of OH^−^ in the wastewater, which promotes the formation of iron hydroxides such as Fe(OH)^2+^ and Fe(OH)^+^, and reacts with SO_4_^2−^ in AMD to form green rust crystals (Equations (1)–(3)).

Fe^2+^ +H_2_O→Fe(OH)^+^ +H^+^ (1)

Fe^3+^ +2H_2_O→Fe(OH)^2+^ +2H^+^ (2)

4Fe(OH)^+^ +2Fe(OH)_2_ + 4OH^−^ + SO_4_^2−^ + 8H_2_O→Fe^II^_4_Fe^III^_2_(OH)_12_SO4·8H_2_O (3)

#### 2.3.2. Fourier Transform Infrared Spectroscopy Analysis

Figure 5 shows the changes in functional groups and chemical bonds in the immobilized particles of dynamic columns 1#, 2#, and 3# before and after adsorption of AMD under specific operating conditions. As shown in Figure 5, a broad and shallow peak in the range of 3455~3300 cm^−1^ corresponds to the stretching vibration of -OH. Peaks at 2920 cm^−1^ and 2856 cm^−1^ are sharp and deep, corresponding to the symmetric and asymmetric stretching vibrations of methyl, methylene, and methylene groups. The peak at 1380 cm^−1^ is sensitive to the structure and is useful for identifying methyl groups. The peak around 1590 cm^−1^ corresponds to the C=O asymmetric stretching vibration, while the peak around 1450 cm^−1^ corresponds to the C-O symmetric stretching vibration. Characteristic peaks representing sulfate and inorganic salts appear at 1110 cm^−1^ and 880 cm^−1^, respectively.

#### 2.3.3. Scanning Electron Microscopy Analysis

Taking dynamic column 3# as an example, scanning electron microscopy (SEM) was performed on Fe^0/2+^-SRB(3#) and adsorbed Fe^0/2+^-SRB(3#), with the results shown in Figure 6a–c. SEM analysis revealed significant differences in the surface morphology of Fe^0/2+^-SRB(3#) before and after the reaction. Before the reaction, the surface of the immobilized particles was smooth and uniform (Figure 6a). After the reaction, the interior of the particles exhibited a flocculent layered structure (Figure 6b), which matches the structural characteristics of sulfate green rust (Fe^II^_4_Fe^III^_2_(OH)_12_SO_4_·8H_2_O) [36]. This sulfate green rust is a layered hydroxide based on Fe (Fe^2+^ and Fe^3+^), with structural features similar to those of layered double hydroxides like hydrotalcite. The surface of the immobilized particles was rough, with irregular pore structures, and SRB appeared around the pores. Additionally, a flaky material around the pores of the immobilized particles (Figure 6c) was observed, which matches the structural characteristics of MnS. The EDS spectrum of adsorbed Fe^0/2+^-SRB(3#) shown in Figure 6d reveals a uniform distribution of Mn and S, indicating the presence of MnS and sulfate green rust. Additionally, absorption peaks for C, O, Fe, Mg, Al, Si, Mn, and S are present in the spectrum shown in Figure 6e. The elemental composition of adsorbed Fe^0/2+^-SRB(3#) is C (10.88 wt%), O (14.73 wt%), Fe (11.56 wt%), Mg (3.03 wt%), Al (12.89 wt%), Si (21.28 wt%), Mn (13.79 wt%), and S (11.84 wt%).

### 2.4. The Treatment Mechanism of AMD by Fe^0/2+^-SRB(3#)

**Figure 7:**
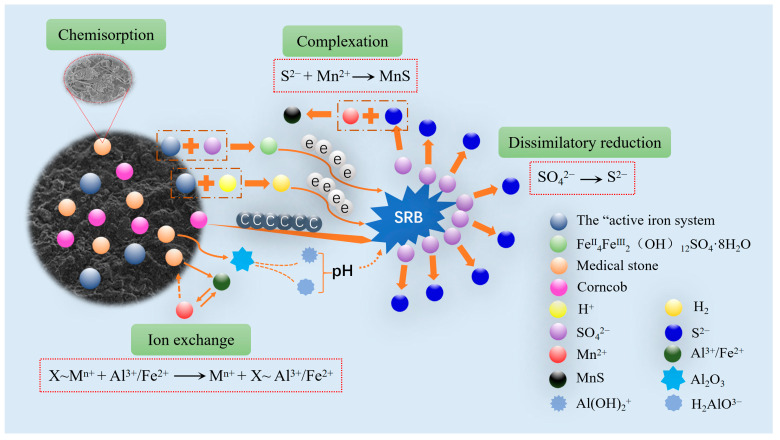
The treatment mechanism of AMD by Fe^0/2+^-SRB(3#).

Fe^0/2+^-SRB(3#) immobilized particles are composed of iron powder, maifan stone, corn cob, and SRB as matrix materials. The interactions among these matrix materials during the remediation of AMD are shown in Figure 7. The iron powder in the waste warm patches exists in an acidic, anaerobic environment as an “active iron” system composed of Fe^0^/Fe^2+^. On one hand, it can directly react with SO_4_^2−^ to form green rust sulfate. Due to the unique interleaved positive and negative charge layers of green rust, which contains a large amount of bound divalent iron, it has high chemical reactivity and a strong electron-donating capacity, making it a suitable electron donor for SRB. On the other hand, it reacts with H^+^ to generate H_2_, which can also serve as an electron donor for SRB in the dissimilatory reduction of SO_4_^2−^. Maifan stone has a loose and porous structure with a large surface area, providing strong chemical adsorption. In aqueous environments, it can release trace elements such as Al^3+^ and Fe^2+^, which can undergo ion exchange with Mn^2+^. The corn cob can decompose nutrients required for the growth and metabolism of SRB, providing a carbon source for the dissimilatory reduction process of SRB [37]. The S^2−^ produced by SRB during the dissimilatory reduction of SO_4_^2−^ complexes with Mn^2+^ on the surface of the immobilized particles, forming MnS. Therefore, in Fe^0/2+^-SRB(3#), there are interactions among the matrix materials, including chemical adsorption, ion exchange, dissimilatory reduction, and surface complexation. Mn^2+^ is primarily removed in the form of the metal sulfide MnS, while Fe^0^/Fe^2+^ can promote the dissimilatory reduction of SO_4_^2−^ by SRB to generate S^2−^. The synergistic interactions among the matrix materials in Fe^0/2+^-SRB(3#) during AMD remediation can increase the pH and reduce the concentrations of heavy metals and sulfates.

### 2.5. Analysis of the Adaptability of Fe^0/2+^-SRB to AMD

#### 2.5.1. Adaptability of Fe^0/2+^-SRB(3#) to Initial pH

**Figure 8:**
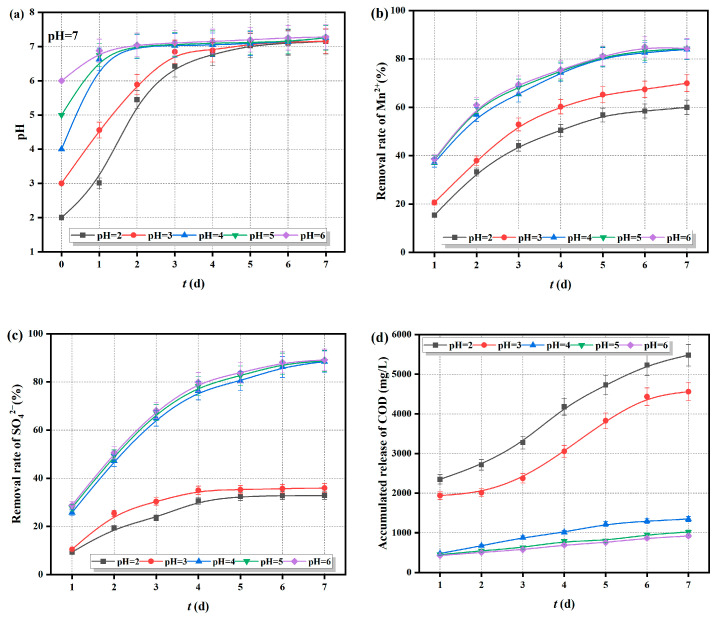
Treatment effect of Fe^0/2+^-SRB(3#) on AMD (at different initial pH values), (**a**) pH, (**b**) removal rate of Mn^2+^, (**c**) removal rate of SO_4_^2−^, (**d**) COD cumulative release.

The Fe^0/2+^-SRB(3#) immobilized particles were added to AMD with initial pH values of 2, 3, 4, 5, and 6, and with Mn^2+^ and SO_4_^2−^ concentrations of 20 mg/L and 800 mg/L, respectively. The changes in pH, Mn^2+^, SO_4_^2−^ concentrations, and chemical oxygen demand (COD) release over time were measured and are shown in Figure 8.

As shown in Figure 8a–d, when the initial pH is greater than 4, the Fe^0/2+^-SRB(3#) immobilized particles raise the pH of each solution to neutral or slightly alkaline within 2 days. The removal rate of Mn^2+^ is relatively high, reaching over 60% within 2 days, while the removal rate of SO_4_^2−^ approaches 80% after 4 days, and the cumulative COD release is around 1000 mg/L after 7 days. When the initial pH is between 2 and 4, the rate of increase of the pH solution is relatively slow, but the improvement effect is obvious on the second day, and it can all reach a neutral or slightly alkaline level within 5 days. It can be seen that the initial pH has a certain impact on the treatment of AMD by the immobilized particles, with a more significant effect when the pH is less than 4. This is because the Fe^0^ in the immobilized particles undergoes corrosion in the acidic mine wastewater, producing Fe^2+^ and forming an “active iron” system. This system converts H^+^ into H_2_, and the generated H_2_ provides an electron donor for SRB. The “active iron” system is also a component of various enzymes in SRB cells, acting as an activator for the enzyme that catalyzes the reduction of SO_4_^2−^. This promotes the dissimilatory reduction of SO_4_^2−^ by SRB, leading to the formation of metal sulfide MnS precipitate as S^2−^ reacts with Mn^2+^. Meanwhile, the maifan stone within the immobilized particles has a pH-regulating effect and, due to its large specific surface area, exhibits strong adsorption properties that can adsorb heavy metal ions in wastewater. However, when the initial pH is low, a large amount of lactate is converted into uncharged lactic acid molecules, which diffuse through the bacterial cell wall and exert toxic effects on SRB. This inhibits the activity of SRB, weakens the dissimilatory reduction of SO_4_^2−^, and reduces the removal rates of Mn^2+^ and SO_4_^2−^. Additionally, the lower the initial pH, the more it promotes the conversion of hemicellulose organic matter in the particles into soluble sugars. This leads to the leakage of the particle matrix, increasing the cumulative COD release.

#### 2.5.2. Adaptability of Fe^0/2+^-SRB(3#) to Initial Concentration of SO_4_^2−^

**Figure 9:**
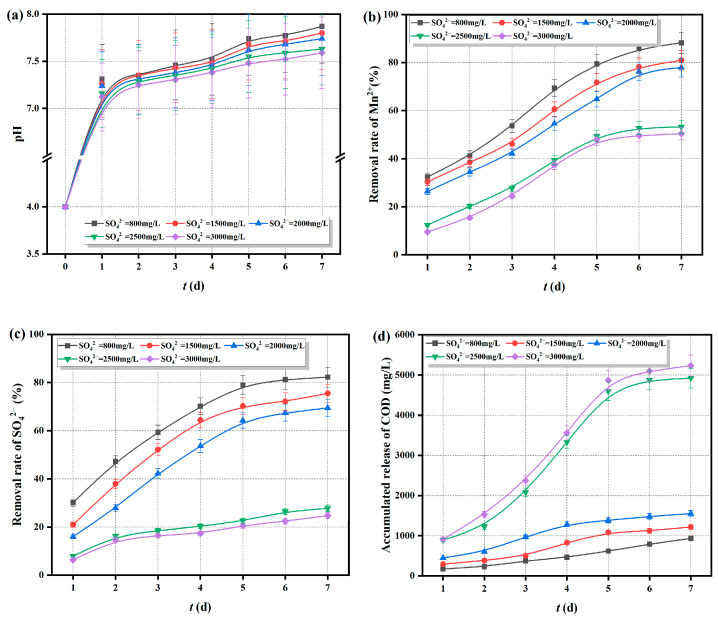
Treatment effect of Fe^0/2+^-SRB(3#) on AMD (at different initial concentration of SO_4_^2−^), (**a**) pH, (**b**) removal rate of Mn^2+^, (**c**) removal rate of SO_4_^2−^, (**d**) COD cumulative release.

The Fe^0/2+^-SRB(3#) immobilized particles were added to AMD with an initial pH of 4, an Mn^2+^ concentration of 20 mg/L, and SO_4_^2−^ concentrations of 800, 1500, 2000, 2500, and 3000 mg/L. The changes in pH, Mn^2+^, SO_4_^2−^ concentrations, and COD release over time were measured and are shown in Figure 9.

As shown in Figure 9a, the pH curves follow a similar trend over time, with the pH rapidly rising to neutral or slightly alkaline levels. This indicates that the initial SO_4_^2−^ concentration has little effect on the ability of Fe^0/2+^-SRB(3#) immobilized particles to increase the pH of the solution. Figure 9b–d shows that when the initial SO_4_^2−^ concentration is less than 2500 mg/L, the removal rate of Mn^2+^ by the immobilized particles is relatively high, reaching nearly 80% after 7 days. The removal rate of SO_4_^2−^ also exceeds 70% after 7 days, with a cumulative COD release of around 1000 mg/L. However, when the initial SO_4_^2−^ concentration is greater than 2500 mg/L, the removal rates of Mn^2+^ and SO_4_^2−^ are lower, while the cumulative COD release is higher, exceeding 5000 mg/L. It is evident that under high SO_4_^2−^ concentrations, the excess H_2_S produced by SO_4_^2−^ reduction can be toxic to SRB, inhibiting SRB activity and weakening the dissimilatory reduction of SO_4_^2−^, thereby reducing the removal rates of Mn^2+^ and SO_4_^2−^. Additionally, the excess H_2_S gas permeates and exits the particles, leading to the leakage of the particle matrix and an increase in the cumulative COD release within the particles.

#### 2.5.3. Adaptability of Fe^0/2+^-SRB(3#) to Initial Concentration of Mn^2+^

**Figure 10:**
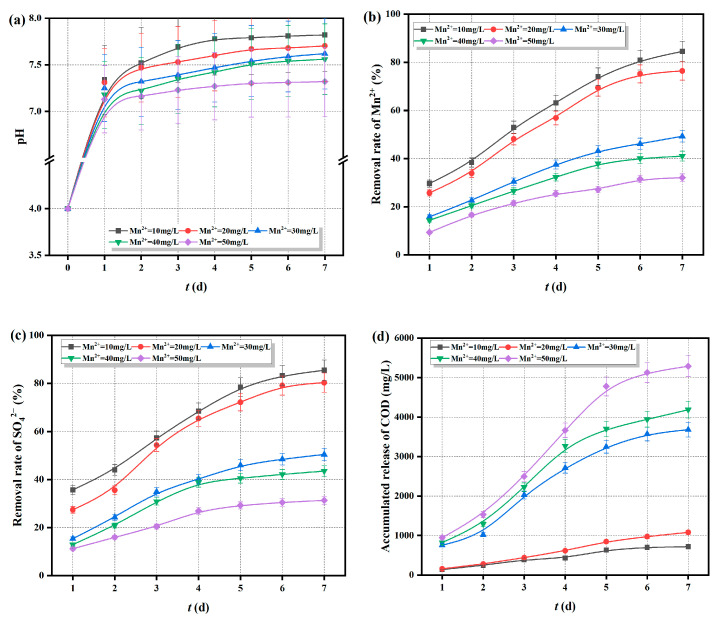
Treatment effect of Fe^0/2+^-SRB(3#) on AMD (at different initial concentrations of Mn^2+^), (**a**) pH, (**b**) removal rate of Mn^2+^, (**c**) removal rate of SO_4_^2−^, (**d**) COD cumulative release.

The Fe^0/2+^-SRB(3#) immobilized particles were added to AMD with an initial pH of 4, an SO_4_^2−^ concentration of 800 mg/L, and Mn^2+^ concentrations of 10, 20, 30, 40, and 50 mg/L. The changes in pH, Mn^2+^, SO_4_^2−^ concentrations, and COD release over time were measured and are shown in Figure 10.

As shown in Figure 10a, the pH curves exhibit a consistent trend over time, rapidly increasing to neutral or slightly alkaline levels. This indicates that the initial Mn^2+^ concentration has little effect on the ability of Fe^0/2+^-SRB(3#) immobilized particles to raise the pH of the solution. Figure 10b–d show that when the initial Mn^2+^ concentration is less than 20 mg/L, the immobilized particles achieve a high removal rate for Mn^2+^, reaching nearly 80% after 7 days. The removal rate of SO_4_^2−^ also exceeds 70% after 7 days, with a cumulative COD release of around 1000 mg/L. However, when the initial Mn^2+^ concentration is greater than 20 mg/L, the removal rates of Mn^2+^ and SO_4_^2−^ are lower, while the cumulative COD release is higher, exceeding 5000 mg/L. It is evident that at high Mn^2+^ concentrations, Mn^2+^ diffuses into the immobilized particles, exerting toxic effects on SRB, reducing SRB activity, and weakening the dissimilatory reduction process. This diminishes the formation of MnS precipitate from S^2−^ and Mn^2+^, leading to reduced removal rates of Mn^2+^ and SO_4_^2−^. Additionally, the intrusion of Mn^2+^ disrupts the structure of the immobilized particles, causing the leakage of the corncob matrix and an increase in cumulative COD release. Although a higher COD/SO_4_^2−^ ratio enhances SRB bioactivity, it cannot counteract the toxic effects of high Mn^2+^ concentrations on SRB. As a result, the efficiency of SRB in the dissimilatory reduction of SO_4_^2−^ remains low.

In summary, Fe^0/2+^-SRB(3#) can adapt to AMD with an initial pH greater than 4, SO_4_^2−^ concentration below 2500 mg/L, and Mn^2+^ concentration below 20 mg/L, making it an excellent adsorbent material for treating AMD.

## 3. Materials and Methods

### 3.1. Materials and Simulated AMD

**Figure 11:**
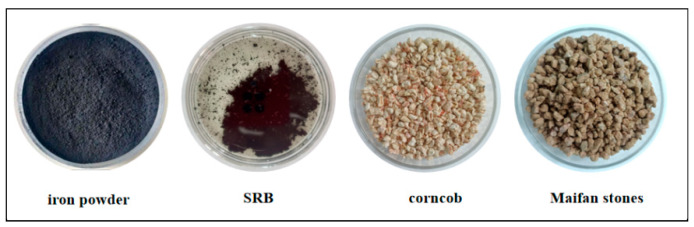
Matrix materials in Fe^0^/Fe^2+^-SRB immobilized particles.

The matrix materials used to prepare the Fe^0^/Fe^2+^-SRB immobilized particles included iron powder, SRB, corn cobs, and maifan stone (Figure 11). The iron powder served as the reduction activation material for the immobilized particles and was sourced from used heat packs discarded by Hunan Aixinyuan Traditional Chinese Medicine Co., Ltd. (Yueyang, China). After normal use, the non-woven fabric packaging was cut open, and the residue was emptied. A magnet was used to separate the iron powder from other powders (e.g., vermiculite, activated carbon, superabsorbent resin). The iron powder was then washed in a 0.5 mol/L HCl solution for 2 h to remove surface oxides and oil, ground manually to a particle size of 48–75 μm, and stored in a brown reagent bottle for later use. The SRB used as the biological strain was sourced from the return sludge of a secondary sedimentation tank at a sewage treatment plant in Fuxin. After filtering out impurities, the SRB was added to a modified Starkey medium and cultured in an anaerobic incubator at a constant temperature of (37 ± 1 °C) for two weeks. When the bottle was opened, the strong smell of rotten eggs indicated successful SRB selection, confirmed by the appearance of a black precipitate when a small amount of the culture was added to FeSO_4_. This indicated that SRB had become the dominant strain in the sludge. The acclimated SRB sludge suspension was centrifuged at 3000 rpm for 10 min, after which the supernatant was discarded, leaving a concentrated sludge with a mass concentration of 500 mg/L, making it ready for use. Corn cobs, serving as the cohesive carbon source within the immobilized particles to provide nutrients for SRB growth and metabolism, were sourced from local farmland in Fuxin. The cobs were sun-dried, mechanically crushed, and processed into particles smaller than 150 μm, making it ready for use. Maifan stone, used as a surface adsorption material, was purchased from a maifan stone sales company in Fuxin, originating from Linyi, Shandong Province. The composition and content of Maifan stone are shown in Table 3. It was processed and ground to the required particle size of 48–75 μm, washed 2–3 times with deionized water to remove impurities and suspended matter, and then dried at 105 °C, making it ready for use.

According to groundwater monitoring results from a mining area in Huludao over the past five years, pH values range from 2.0 to 6.0, Mn^2+^ concentrations range from 1.0 to 5.0 mg/L (occasionally exceeding 10 mg/L), and SO_4_^2−^ concentrations range from 800 to 2500 mg/L. According to the “Standards for Drinking Water Quality (GB5749-2006)” [38], the pH limit is 6.5 to 8.5, with concentration limits for manganese and sulfate set at 0.1 mg/L and 250 mg/L, respectively. Therefore, the groundwater in this mining area is classified as typical AMD, characterized by high acidity, high concentrations of heavy metals, and high sulfate levels—commonly referred to as the “three highs”. Considering the complexity of actual AMD, the experimental water samples were prepared to simulate the groundwater characteristics of this mining area.

According to the results of previous studies [19,39], the removal rate of Mn^2+^ is relatively low when the biological method is used to repair AMD, so the main pollutant selected in the experiment is Mn^2+^.

### 3.2. Preparation of Fe^0^/Fe^2+^-SRB Immobilized Particles

**Figure 12:**
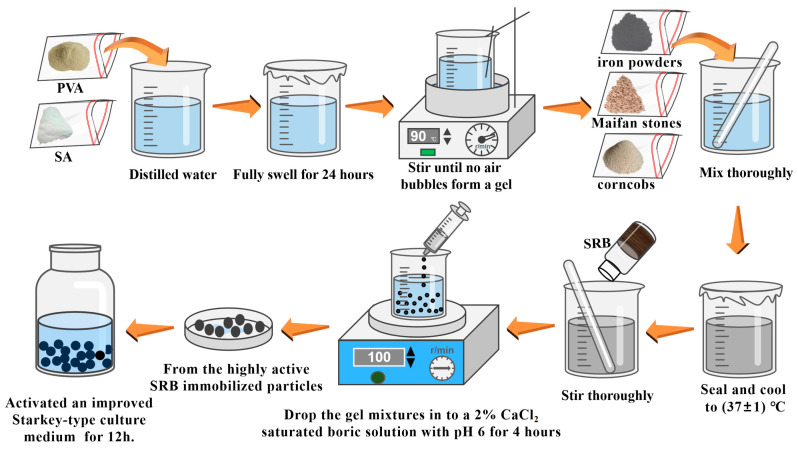
Preparation process of high activity immobilized particles.

Polyvinyl alcohol (PVA) and sodium alginate (SA) were dissolved in 100 mL of distilled water, the mixture was sealed at room temperature, and allowed to swell fully for 24 h. Then, it was placed in a 90 °C constant-temperature water bath and stirred until a bubble-free gel was formed. Gradually a certain amount of iron powder, corn cobs, and maifan stone were added into the gel in sequence, stirred thoroughly, and then removed from the mixture. It was sealed and cooled to (37 ± 1 °C). A certain amount of SRB sludge was added to the prepared gel and stirred evenly. A specific syringe was used to drop the gel mixture into a 2% CaCl_2_-saturated boric acid solution with a pH of 6, forming immobilized particles. Crosslinking was carried out using a six-station stirrer at a stirring speed of 100 r/min. After 4 h, the particles were removed, rinsed with 0.9% saline solution, and then the surface moisture was blotted dry; this process was repeated three times. Before use, the particles were activated for 12 h in an anaerobic environment using a modified Starkey medium solution without organic components. The preparation process of Fe^0^/Fe^2+^-SRB immobilized particles is shown in Figure 12.

This experiment aims to explore the synergistic enhancement effect of Fe^0^/Fe^2+^ on SRB-immobilized particles. Based on the presence or absence of both Fe^0^/Fe^2+^ and SRB within the immobilized particles, three types of immobilized particles with different matrix compositions were prepared. The matrix compositions are shown in Table 4. Nitrogen adsorption–desorption experiments were conducted on the three types of immobilized particles to investigate their pore structure characteristics (Table 4). It was found that the specific surface area, average pore volume, and average pore diameter of Fe^0^/Fe^2+^-SRB(3#) were all greater than those of Fe^0^/Fe^2+^-SRB(1#) and Fe^0^/Fe^2+^-SRB(2#). Consequently, Fe^0^/Fe^2+^-SRB(3#) has more adsorption sites, which helps to reduce surface blockage and facilitates the adsorption of more Mn^2+^ on the particle surface.

### 3.3. Dynamic Experiments

**Figure 13:**
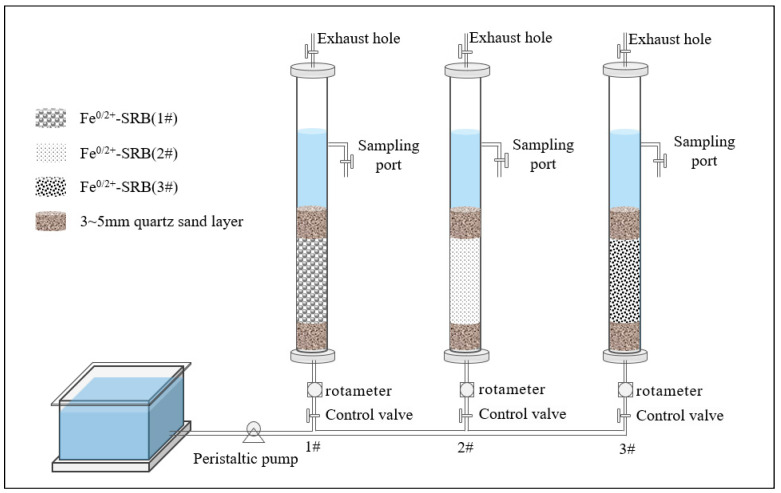
Schematic diagram of the dynamic experimental column setup.

The dynamic experiments were conducted under nearly constant temperature (25 ± 1 °C) and constant humidity (45% ± 2%) conditions. Based on the operational principle of a constant-speed upflow expanded bed filter, three anaerobic, upflow, continuously operating dynamic experimental columns were constructed (Figure 13). To prevent the inlet from clogging, gauze was placed at the bottom of the dynamic column. To evenly distribute the flow and prevent the loss of immobilized particles, quartz sand layers were set above and below the immobilized particle layer. To minimize potential wall effects and axial dispersion in the fixed bed column, the bed length-to-particle diameter ratio was maintained above 20. Therefore, organic glass columns with an inner diameter of 55 mm and a height of 500 mm were selected for the dynamic columns. The materials inside the column, from bottom to top, were as follows: 5 mm of gauze, 50 mm of quartz sand with a particle size of 3–5 mm, a specific height of Fe^0^/Fe^2+^-SRB immobilized particle adsorption layer, and 50 mm of quartz sand with a particle size of 3–5 mm. The adsorption layer in dynamic Column 1# consisted of Fe^0^/Fe^2+^-SRB (1#), in dynamic column 2# of Fe^0^/Fe^2+^-SRB (2#), and in dynamic column 3# of Fe^0^/Fe^2+^-SRB (3#). The inflow method for the dynamic columns was bottom-in and top-out, with a pore diameter of 2 mm. Wastewater was pumped through a peristaltic pump, and a glass rotor flowmeter controlled the flow rate and velocity. Before the experiment, an anaerobic environment was achieved by displacing air with deionized water. During the experiment, wastewater filled the adsorption layer voids, maintaining a liquid level of more than 50 mm above the upper quartz sand layer. Samples were taken at 8 h intervals at the sampling port, and the Mn^2+^ concentration in both the raw and treated water was measured. The experiment continued until the Mn^2+^ concentration in the treated water stabilized at the adsorption breakthrough point. The study analyzes the influence of different adsorption layer heights (150, 200, 250 mm), inflow velocities (1 × 10^−5^, 5 × 10^−5^, 10 × 10^−5^ m^3^/s), and initial Mn^2+^ concentrations (2, 5, 10 mg/L) on the Mn^2+^ adsorption process by Fe^0^/Fe^2+^-SRB.

### 3.4. Breakthrough Curve of Mn^2+^ Adsorption by Fe^0/2+^-SRB

The breakthrough curve is plotted based on the ratio of the outflow concentration of Mn^2+^ (*C*_t_) to the inflow concentration (*C*_0_) as a function of operating time (*t*) [40]. The outflow concentration of Mn^2+^ reaching 5% of the inflow concentration (*C*_t_/*C*_0_ = 0.05) is defined as the breakthrough point, while the outflow concentration reaching 95% of the inflow concentration (*C*_t_/*C*_0_ = 0.95) is defined as the exhaustion point. The corresponding times are termed the breakthrough time (*T*_b_) and exhaustion time (*T*_e_), respectively. The total ion adsorption (*q*_total_) and adsorption capacity (*q*_e_) of the dynamic column are given by Equations (4) and (5), respectively.

(4) $$q_{\mathrm{total}}=\frac{Q}{1000}\int_{0}^{t}\left(C_{0}-C_{t}\right)d_{t}$$

(5) $$q_{e}=\frac{q_{\mathrm{total}}}{m}$$

where *Q* is the inflow volumetric flow rate (m^3^/s), and *m* is the mass of the high activity immobilized particles (g).

### 3.5. Dynamic Adsorption Kinetics Models of Mn^2+^ by Fe^0/2+^-SRB

The dynamic adsorption process of Mn^2+^ by high activity SRB immobilized particles is analyzed using the Bohart–Adams and Thomas models [41,42], as shown in Equations (6) and (7). The Bohart–Adams model is based on surface reaction theory, assuming that adsorption equilibrium is not instantaneous and that the relationship between the concentration and position of adsorbates in the adsorption layer is linear. It evaluates the adsorption performance of immobilized particles for Mn^2+^ in the initial adsorption phase using the mass transfer rate constant *k*_Th_ and the ion adsorption saturation concentration *N*_0_. The Thomas model is based on Langmuir adsorption–desorption theory, assuming no axial dispersion. It evaluates the adsorption performance of immobilized particles for Mn^2+^ under different column heights, velocity, and initial concentrations using the mass transfer rate constant *k*_Th_ and the maximum adsorption capacity *q*_0_.

(6) $$\ln\left(\frac{C_{t}}{C_{0}}\right)=k_{\mathrm{AB}}C_{0}t-k_{\mathrm{AB}}N_{0}\left(\frac{H}{W}\right)$$

(7) $$\ln\left(\frac{C_{0}}{C_{t}}-1\right)=\frac{k_{\mathrm{Th}}q_{0}m_{1}}{Q}-k_{\mathrm{Th}}C_{0}t$$

where *k*_AB_ is the Bohart–Adams mass transfer rate constant (L/(mg·min)); *N*_0_ is the adsorption saturation concentration of Mn^2+^ (mg/L); *H* is the height of the dynamic column (cm); and *W* is the solution flow rate (cm/min). *k*_Th_ is the Thomas mass transfer rate constant (L/(mg·min)); *q*_0_ is the maximum adsorption capacity of the immobilized particles (mg/g); and *m*_1_ is the mass of the immobilized particles in the dynamic column (g).

### 3.6. Adaptability of Fe^0/2+^-SRB to AMD

To evaluate the adaptability of Fe^0/2+^-SRB immobilized particles to different AMD environments, batch experiments were conducted. The prepared Fe^0/2+^-SRB immobilized particles (20 g) were placed in AMD solutions with varying initial pH, Mn^2+^, and SO_4_^2−^ concentrations at a solid-to-liquid ratio of 1:10 (m, g/mL). These were anaerobically sealed in 250 mL stoppered Erlenmeyer flasks and stirred on a thermostatic magnetic stirrer at 30 °C and 100 rpm. The pH, Mn^2+^, SO_4_^2−^ concentrations, and COD release in the solution were measured to analyze the adaptability of the Fe^0/2+^-SRB immobilized particles to AMD under different initial conditions. pH values were adjusted to 2, 3, 4, 5, and 6 using H_2_SO_4_ solutions. SO_4_^2−^ and Mn^2+^ concentrations were adjusted to 800, 1500, 2000, 2500, and 3000 mg/L and 10, 20, 30, 40, and 50 mg/L, respectively, using Na_2_SO_4_ and MnSO_4_.

### 3.7. Detection Items and Methods

The concentration of Mn^2+^ was determined using the potassium iodate spectrophotometric method; the concentration of SO_4_^2−^ was determined using the barium chromate spectrophotometric method. The phase composition of Fe^0/2+^-SRB was characterized using a Shimadzu (Tokyo, Japan) XRD-6100 X-ray diffractometer, while the microscopic morphology of Fe^0/2+^-SRB was observed using a Hitachi (Tokyo, Japan) S-3400N scanning electron microscope. The specific surface area and pore size of Fe^0/2+^-SRB were analyzed using a ASAP2020 Conta Atuosorb-iQ specific surface area and porosity analyzer manufactured by Micromeritics (Shanghai, China), and the chemical bonds and functional group changes in Fe^0/2+^-SRB were characterized using an IRPrestige-21 Fourier-transform infrared spectrometer manufactured by Shimadzu Corporation (Tokyo, Japan).

## 4. Conclusions

In this study, a highly adsorptive Fe^0/2+^-SRB(3#) was prepared using microbial immobilization technology for the remediation of AMD.

(1) When the adsorption layer height was 200 mm, the inflow rate was 5 × 10^−5^ m^3^/s, and the initial Mn^2+^ concentration was 10 mg/L, the dynamic adsorption capacity for Mn^2+^ in the constructed dynamic columns was maximized.
(2) Compared with dynamic columns 1# and 2#, dynamic column 3# showed the best performance in treating AMD, indicating that the presence of the Fe^0^/Fe^2+^ composite system enhances the remediation capability of Fe^0/2+^-SRB. Additionally, the Thomas model accurately describes the adsorption kinetics of Mn^2+^ by Fe^0/2+^-SRB(3#).
(3) In Fe^0/2+^-SRB(3#), there are interactions of chemical adsorption, ion exchange, dissimilation–reduction reaction, and surface complexation among the substrates. Mn^2+^ is primarily removed in the form of manganese sulfide (MnS), while Fe^0^/Fe^2+^ facilitates the dissimilatory reduction of SO_4_^2−^ by SRB, leading to the generation of S^2−^.
(4) Fe^0/2+^-SRB(3#) can adapt to AMD with an initial pH greater than 4, SO_4_^2−^ concentration below 2500 mg/L, and Mn^2+^ concentration below 20 mg/L, making it an excellent adsorbent material for treating AMD.

## Figures and Tables

**Table 1:** Adsorption mass transfer parameters of Fe^0/2+^-SRB for Mn^2+^ at different concentrations.

Column	Different Adsorption Layer Heights			Different Inflow Velocity			Different Initial Concentrations		
	*H* (mm)	*Q*_total_ (mg)	*Q*_e_ (mg·g^−1^)	*Q* (m^3^·s^−1^)	*Q*_total_ (mg)	*Q*_e_ (mg·g^−1^)	*C*_0_ (mg·L^−1^)	*Q*_total_ (mg)	*Q*_e_ (mg·g^−1^)
1#	150	1592.324 ± 79.616	5.3077 ± 0.2654	1 × 10^−5^	2015.303 ± 100.765	4.2879 ± 0.2144	2	258.265 ± 12.931	0.5495 ± 0.0275
	200	1974.233 ± 78.712	4.2005 ± 0.2100	5 × 10^−5^	3686.187 ± 184.309	7.8430 ± 0.3922	5	561.209 ± 28.060	1.1941 ± 0.0597
	250	2387.693 ± 119.385	3.9143 ± 0.1957	10 × 10^−5^	2634.256 ± 131.713	5.6048 ± 0.2802	10	1002.388 ± 50.119	2.1327 ± 0.1066
2#	150	1086.530 ± 54.327	3.6218 ± 0.1181	1 × 10^−5^	1233.536 ± 61.677	2.6245 ± 0.1312	2	219.335 ± 10.967	0.4667 ± 0.0233
	200	1523.578 ± 76.179	3.2417 ± 0.1621	5 × 10^−5^	2238.492 ± 111.925	4.7627 ± 0.2381	5	463.763 ± 23.188	0.9867 ± 0.0493
	250	1787.183 ± 89.359	2.9298 ± 0.1465	10 × 10^−5^	1883.551 ± 94.178	4.0076 ± 0.2004	10	755.581 ± 37.779	1.6076 ± 0.0804
3#	150	1958.153 ± 79.908	6.5272 ± 0.3264	1 × 10^−5^	2017.284 ± 100.864	4.2921 ± 0.2146	2	325.597 ± 16.280	0.6928 ± 0.0346
	200	2452.581 ± 122.629	5.2183 ± 0.2609	5 × 10^−5^	4120.802 ± 206.040	8.7677 ± 0.4384	5	785.711 ± 39.286	1.6717 ± 0.0836
	250	3068.945 ± 153.447	5.0311 ± 0.2516	10 × 10^−5^	2719.803 ± 135.990	5.7868 ± 0.2893	10	1365.539 ± 68.277	2.8863 ± 0.1443

**Table 2:** Fitting parameters of the Bohart–Adams and Thomas models for Fe^0/2+^-SRB adsorption of Mn^2+^.

Fitting Parameters			Bohart–Adams Model			Thomas Model		
*H* (mm)	*Q* (m^3^·s^−1^)	*C*_0_ (mg·L^−1^)	*k*_AB_ (L·mg^−1^·min^−1^)	*N*_0_ (mg·L^−1^)	*R* ^2^_AB_	*k*_Th_ (L·mg^−1^·min^−1^)	*q*_0_ (mg·g^−1^)	*R* ^2^_Th_
150	1 × 10^−5^	10	8.341 × 10^−6^	4.086	0.914	3.314 × 10^−6^	6.554	0.989
200	1 × 10^−5^	10	7.254 × 10^−6^	3.947	0.923	3.105 × 10^−6^	5.252	0.978
250	1 × 10^−5^	10	5.247 × 10^−6^	3.214	0.955	2.494 × 10^−6^	5.055	0.991
200	1 × 10^−5^	10	5.264 × 10^−6^	3.524	0.825	2.483 × 10^−6^	4.320	0.985
200	5 × 10^−5^	10	6.584 × 10^−6^	8.259	0.893	3.096 × 10^−6^	9.054	0.994
200	10 × 10^−5^	10	5.748 × 10^−6^	6.058	0.875	2.785 × 10^−6^	5.783	0.986
200	1 × 10^−5^	2	8.547 × 10^−6^	0.351	0.904	19.844 × 10^−6^	0.702	0.995
200	1 × 10^−5^	5	7.458 × 10^−6^	1.458	0.897	6.623 × 10^−6^	1.672	0.994
200	1 × 10^−5^	10	6.251 × 10^−6^	2.225	0.910	4.963 × 10^−6^	2.875	0.988

**Table 3:** The composition and content of Maifan stone.

Producing Area	Composition and Content of Maifan Stone (%)										
	SiO_2_	Al_2_O_3_	Na_2_O	Fe_2_O_3_	CaO	K_2_O	MgO	TiO_2_	MnO	P_2_O_5_	Reference
Shandong Province, Linyi	67.90	15.75	5.45	2.82	2.51	1.59	0.66	0.32	0.06	0.056	[33]
Shandong Province, Mengyin	66.97	16.00	5.52	2.80	1.66	2.70	1.40	—	—	—	[34]

**Table 4:** Composition and pore structure characteristics of Fe^0^/Fe^2+^-SRB immobilized particles.

Sample	Matrix Content (g)				Pore Structure Characteristics		
	SRB	Iron Powder	Corncobs	Maifan Stones	Average Pore Volume (cm^3^·g^−1^)	Average Pore Diameter (nm)	Specific Surface Area (m^2^·g^−1^)
Fe^0/2+^-SRB(1#)	3.0	0.0	0.5	0.3	0.0153	7.8325	11.0725
Fe^0/2+^-SRB(2#)	0.0	0.6	0.0	0.3	0.0069	5.7564	9.5360
Fe^0/2+^-SRB(3#)	3.0	0.6	0.5	0.3	0.0325	10.3327	15.3420

## Data Availability

All the data have been included in the study.
